# Enhancing machine learning-based sentiment analysis through feature extraction techniques

**DOI:** 10.1371/journal.pone.0294968

**Published:** 2024-02-14

**Authors:** Noura A. Semary, Wesam Ahmed, Khalid Amin, Paweł Pławiak, Mohamed Hammad

**Affiliations:** 1 Department of Information Technology, Faculty of Computers and Information, Menoufia University, Shibin El Kom, Egypt; 2 Department of Information Technology, Faculty of Computers and Artificial Intelligence, South Valley University, Hurghada, Egypt; 3 Department of Computer Science, Faculty of Computer Science and Telecommunications, Cracow University of Technology, Krakow, Poland; 4 Institute of Theoretical and Applied Informatics, Polish Academy of Sciences, Gliwice, Poland; 5 EIAS Data Science Lab, College of Computer and Information Sciences, Prince Sultan University, Riyadh, Saudi Arabia; Bahria University - Lahore Campus, PAKISTAN

## Abstract

A crucial part of sentiment classification is featuring extraction because it involves extracting valuable information from text data, which affects the model’s performance. The goal of this paper is to help in selecting a suitable feature extraction method to enhance the performance of sentiment analysis tasks. In order to provide directions for future machine learning and feature extraction research, it is important to analyze and summarize feature extraction techniques methodically from a machine learning standpoint. There are several methods under consideration, including Bag-of-words (BOW), Word2Vector, N-gram, Term Frequency- Inverse Document Frequency (TF-IDF), Hashing Vectorizer (HV), and Global vector for word representation (GloVe). To prove the ability of each feature extractor, we applied it to the Twitter US airlines and Amazon musical instrument reviews datasets. Finally, we trained a random forest classifier using 70% of the training data and 30% of the testing data, enabling us to evaluate and compare the performance using different metrics. Based on our results, we find that the TD-IDF technique demonstrates superior performance, with an accuracy of 99% in the Amazon reviews dataset and 96% in the Twitter US airlines dataset. This study underscores the paramount significance of feature extraction in sentiment analysis, endowing pragmatic insights to elevate model performance and steer future research pursuits.

## 1. Introduction

Public opinion plays a significant role in business operations and product perception. Additionally, since it explains human behaviour and how other people’s opinions affect it, public opinion analysis is very helpful to governments. The application of sentiment analysis holds significant value in discerning the sentiment and perspective expressed in textual material [1–3]. The problem can be framed as either a binary or multi-class classification task. Binary sentiment analysis separates texts into positive and negative classes, while multiclass sentiment analysis separates them into fine-grained categories [4, 5]. Sentiment analysis can be done on social media platforms like Twitter and websites, including comments, forums, blogs, and microblogs. The analysis of sentiment is usually performed by applying a rule-based system or a machine learning system. In recent years, machine learning systems have become increasingly popular because they are more versatile and easier to apply than traditional rule-based systems. Machine learning algorithms are trained to recognize underlying patterns in documents in order to classify them [6, 7]. Sentiment analysis based on machine learning involves three steps: feature extraction, feature selection, and machine learning classifier. The selection of feature extraction for better outcomes in many natural language processing (NLP) tasks, especially in sentiment analysis, is vital [8, 9]. Feature extraction is the methodological procedure of identifying and converting pertinent material from its original form into a more succinct and significant representation, with the purpose of facilitating analysis [10, 11].

One of the major challenges in sentiment classification tasks is the choice of feature extraction technique. In the analysis process, features are represented as a single unit and used to classify documents into the corresponding polarities [12, 13]. As a result of the large number of features, the overall system will be impacted by a heavy processing load, and the use of irrelevant features produces overfitting or underfitting models of classifiers. The system’s performance is optimal when the feature set is considerably small but informative and accurate. Text embeddings or feature extraction techniques map text data into vectors, which can be a set of real numbers (a vector) that can be used as input to a machine learning model. There are numerous word representation models now in use. Based on the word distribution data, the models can be categorized as either traditional models or static models, according to [14, 15]. Depending on the specific feature extraction technique used, different types of information can be extracted from the text data. This study offers the following research points:

1. The use of different techniques for feature extraction is investigated for sentiment analysis tasks and provides a useful resource for assessing the strengths and limitations of different feature extraction approaches and making informed choices.
2. We explore the exact relationship between feature extraction, classification performance, and the training time of the methods.
3. Providing a discussion on how the accuracy of the machine learning algorithms changes with different feature extractions. It is still an unsolved problem and an unanswered question on how to select a suitable feature extraction technique to be used to obtain the best performance for capturing sentiment in different social media datasets.
4. In this study, we uncover which feature extraction technique is most effective for sentiment analysis tasks as well as the implications of our findings for practical applications such as monitoring social media sites, among other areas.

As for the rest of the study, it is arranged as follows: The background literature section presents relevant sentiment analysis work. The proposed system section explains the methodology and details of the study. The experimental results section displays the results. In discussion section, the outcomes are discussed. The conclusion section includes a summary of the paper’s findings and future work.

## 2. Background literature

This section reviews relevant studies on feature extraction for sentiment analysis using machine learning models.

### 2.1 Previous studies

For sentiment analysis tasks, most of the feature extraction algorithms have been used with different machine learning models, but few studies have looked at their impact on their performance. A comparative study of sentiment analysis is shown in Table 1. Comparing and contrasting prior research will enable the present study to explore a different area that has not been discussed previously. The n-gram and term frequency-inverse document frequency (TF-IDF) are widely recognized as prominent feature extraction strategies in machine learning models, as seen by their prevalence in numerous prior studies.

**Table 1:** Literature survey of sentiment analysis.

Ref.	Dataset	Feature Extraction	Model	Results
Ahmed and Ahmed [16]	Collected news articles	TF-IDF	NB	Accuracy = 89.30%
Gaur et al. [17]	Twitter sentiment 140	TF-IDF	NB	Accuracy = 84.44%
Qi and Shabrina [18]	Collected tweets about COVID-19	TF-IDF, and Word2Vec	MNB, SVC, RF, Vader, and Textblob	SVC with TF–IDF outperforms others with accuracy = 71%
Al sari et al. [19]	Instagram, Snapchat, and Twitter datasets	Unigrams	MLP, NB, RF, SVM, and voting	NB algorithm in Twitter with Over-sampling technique achieves accuracy = 85.26%
Mukherjee et al. [20]	Amazon reviews	TF-IDF	MNB, SVM, and ANN	ANN + Negation classifier performs the best with accuracy = 96.32%
Noori [21]	Customer reviews	TF-IDF	NB, SVM, DT, and KNN	Best accuracy reported for DT = 98.9%
Zahoor and Rohilla [22]	Collected tweets about different events	N-gram	NB, SVM, RF, and LSTM	NB outperforms others on most datasets with accuracy = 96.8%
Samuel et al. [23]	COVID-19 tweets	N-gram	NB and LR	NB outperforms LR with accuracy = 91.43%
Kumar et al. [24]	Book reviews	BOW, and Word2Vec	NB, ME, and SVM	SVM has the highest accuracy = 78%
Zarisfi et al. [25]	Twitter datasets	TF-IDF	SVM, MNB and hybrid algorithm	The hybrid method yields a better classification with accuracy = 85.92%

Ahmed and Ahmed [16] applied TF-IDF, random forest (RF), Naïve Bayes (NB), and feature extraction to the collected fake news articles to classify them into positive and negative sentiments. Among the individual classifiers, the NB was the best and achieved the highest accuracy (89.30%).

Gaur *et al*. [17] used a machine learning algorithm based on the NB Classifier with TF-IDF feature extraction to classify the Twitter sentiment 140 dataset. Based on performance metrics including precision, recall, and accuracy, the suggested model’s results showed improved accuracy (84.44%) and precision.

Qi and Shabrina [18] extracted data relating to COVID-19 from Twitter users in England’s major cities. This study compares machine learning models as its main objective, such as multinominal Naïve Bayes (MNB), RF, and support vector classification (SVC), with lexicon-based approaches such as Vader and Textblob using two feature extraction methods (Word2Vec embedding and TF-IDF). Overall, the SVC with TF-IDF had better accuracy than the other models.

Al sari *et al*. [19] created three different datasets from social media platforms to analyze the impressions about Saudi cruises. The methodology of the study is performed by applying machine learning algorithms such as multilayer perceptron (MLP), NB, voting, SVM, RF, and the n-grams feature extraction technique. The RF algorithm achieved 100% classification accuracy with oversampled Snapchat data.

Mukherjee *et al*. [20] presented a customized algorithm for detecting explicit negation. Different machine learning algorithms, such as NB, SVM, and Artificial Neural Networks (ANN), were performed on Amazon reviews to analyze the sentiments. The methodology of TF-IDF was employed to extract features. The ANN with a negative classifier achieved the best accuracy (96.32%).

Noori [21] proposed a new approach to classify customer sentiments. The paper collected customer reviews from an international hotel. The reviews are processed, and then the TF-IDF extractor is applied to build the document vectors and then trained into SVM, ANN, NB, k-nearest neighbor (K-NN), decision tree (DT), and C4.5 models. The result of the DT model is an accuracy of 98.9% with the number of features (1800), and this model performed better than others.

Zahoor and Rohilla [22] used NB, SVM, long short-term memory networks (LSTM), and RF classifiers and compared the findings. The N-gram extraction was used after preprocessing the datasets. The NB model has the highest accuracy on most datasets, such as the BJP and ML Khattar datasets. Samuel et al. [23] used NB and logistic regression (LR) models on tweets about COVID-19. The tweets are transformed into a text corpus, and then the most frequent words are identified using N-grams. Their results indicated a high accuracy of 91% with the NB method and an accuracy of 74% with LR for short tweets, and longer tweets performed relatively worse for both models.

Kumar *et al*. [24] examined how gender and age affected the customer reviews that had been gathered. Maximum entropy (ME), SVM, and LSTM models are applied. The NB, ME, and SVM algorithms all employ the Bag of Words (BOW) feature extraction, while word2vec is used in the LSTM model. The best accuracy for female data is with a group over 50.

Zarisfi *et al*. [25] used SVM and MNB with TF-IDF extraction on four Twitter datasets, namely the Strict Obama-McCain Debate dataset, the Obama-McCain Debate dataset, the STS-Gold dataset, and the Stanford testing dataset. Semantic scoring based on tweet class, semantic similarity, SWN scoring, and TF-IDF methods have been suggested for representing the features in the vector space. In three datasets, the proposed method outperformed the MNB algorithm. The MNB algorithm performs the best of all methods in the STS dataset.

### 2.2 Gap in literature

Previous studies indicate that there is a paucity of literature that needs to be discussed. There is a limited range of techniques used for feature extraction in previous works. The aim of this paper is to address this gap by evaluating different feature extraction techniques on the same dataset when using sentiment classification to choose the most suitable method. We want the best possible results when doing classification, so the method that we choose for feature extraction is important.

## 3. Proposed system

**Fig 1:**
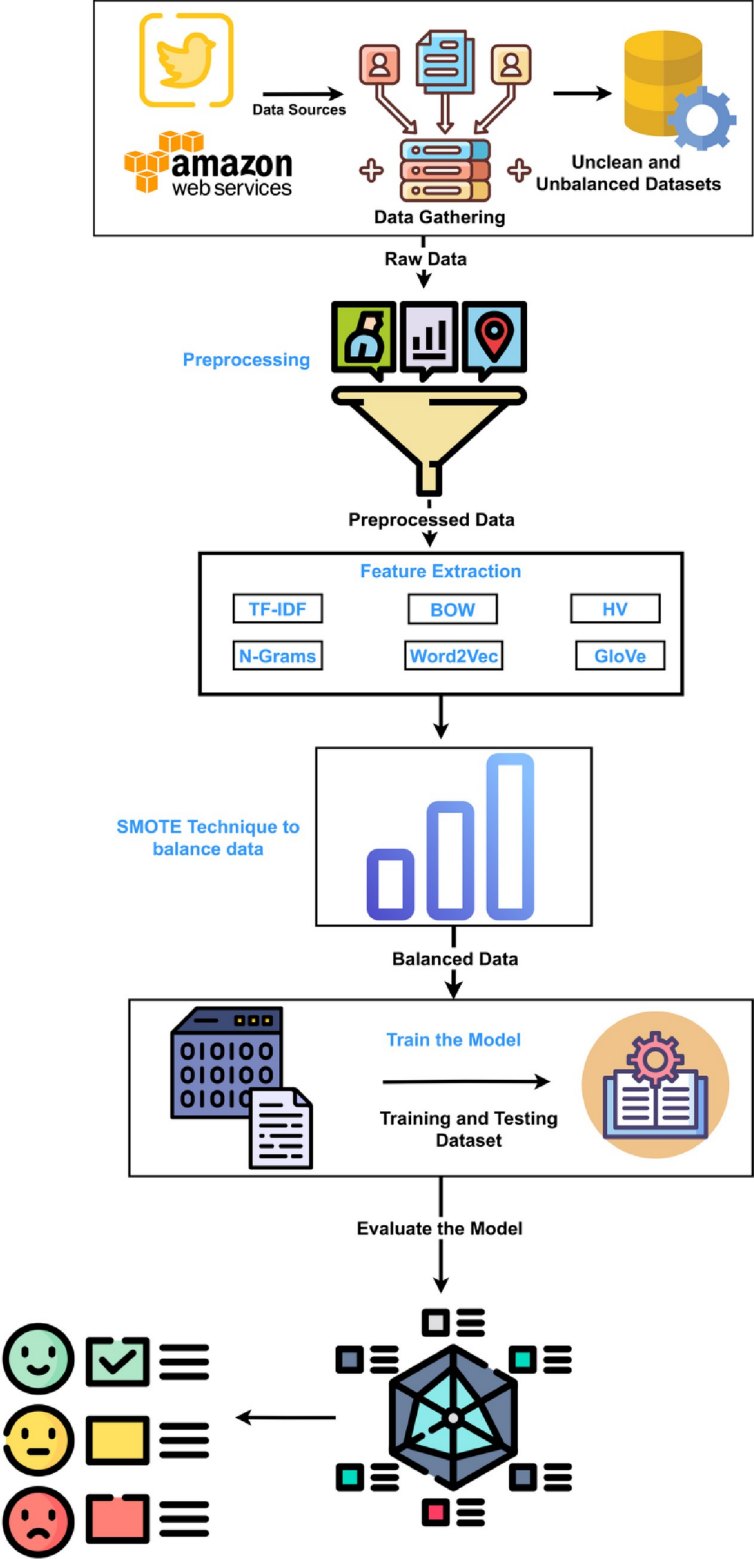
The architecture of the proposed model for sentiment classification.

This section addresses the description of the datasets and preprocessing steps, as well as the feature extraction techniques, the SMOTE technique, and the classification model. Fig 1 illustrates the architectural design of our experiment, while Algorithm 1 provides a summary of our proposed system.

**Algorithm 1.** Framework of Our Proposed System.

Input: Raw dataset D

Output: Sentiment (Positive or Negative or Neutral)

Begin

 Clean input D (remove special symbols, stop-words, emoji, URL, tokenization, etc)

 Assign sentiment labels to D

 Apply feature extraction to transform D into a feature vector

 Apply the SMOTE technique to balance the dataset

 Classify D using the random forest model

End

### 3.1 Dataset description

**Fig 2:**
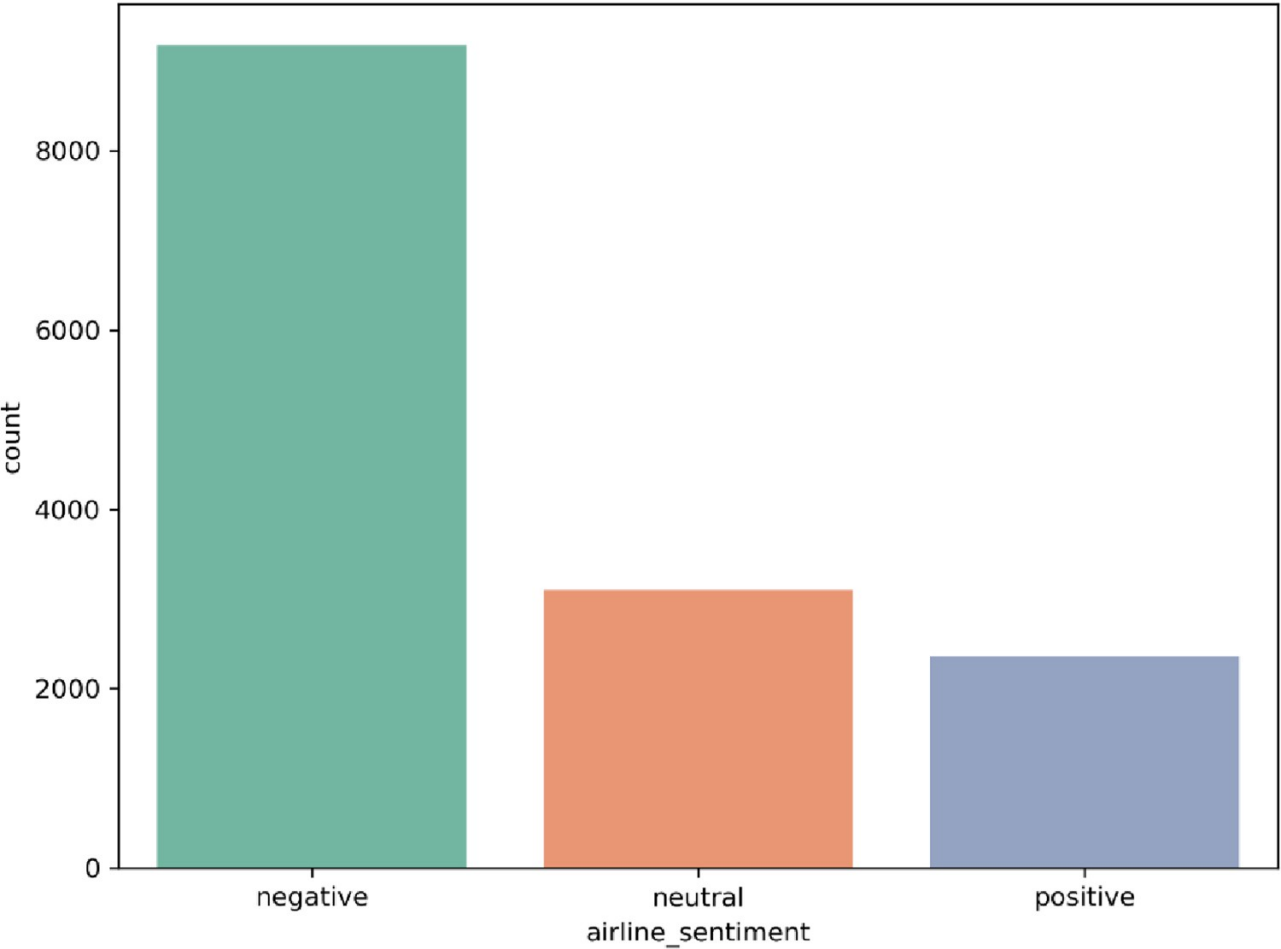
Sentiment data distribution of the Twitter dataset.

**Fig 3:**
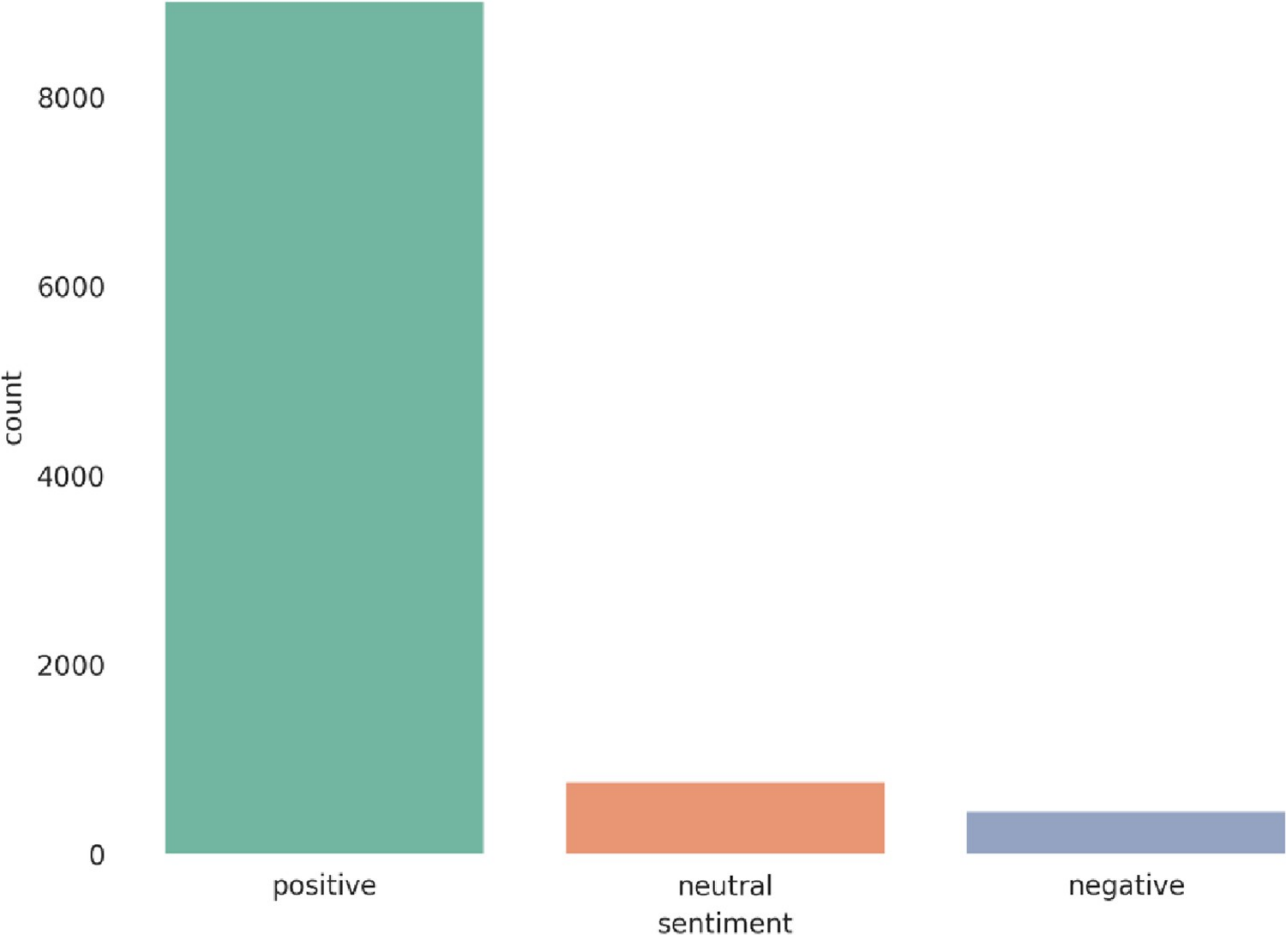
Sentiment data distribution of the Amazon dataset.

For the experiments, we picked two different datasets that consist of real-world user feedback, reflecting the opinions and sentiments of actual customers and users, making them a highly demanded source for researchers in the field. The first dataset is Twitter US Airlines, which CrowdFlower created in 2017. It offers a comprehensive collection of customer reviews of six significant American airlines and contains various features, as shown in Table 2. It has 14640 instances, out of which 2363 are positive tweets, 9178 are negative tweets, and the remaining 3099 are neutral tweets [26]. The second dataset is Amazon musical instrument reviews collected in 2020, which offer a rich collection of customer feedback and contain various features, as shown in Table 3. It has 10261 instances out of which 9022 are positive reviews, 467 are negative reviews, and the remaining 772 are neutral reviews [27]. The datasets are available at https://www.kaggle.com/datasets/crowdflower/twitter-airline-sentiment and https://www.kaggle.com/datasets/eswarchandt/amazon-music-reviews. It can be observed that the two datasets have imbalanced data. Figs 2 and 3 illustrate the distribution of sentiment classes in the datasets.

**Table 2:** Feature description of the Twitter US airlines dataset.

Features	Description
Airline Sentiment Confidence	A numerical attribute that quantifies the amount of confidence in the classification of a tweet into one of three distinct classes.
Negative Reason	The rationale for deeming this tweet as having a negative connotation.
Negative Reason Confidence	The degree of certainty in establishing the underlying cause of a negative tweet.
Airline	The airline company’s name.
Retweet Count	The quantification of retweets received by a specific tweet.
Text	Tweet initially published by the user.
Airline Sentiment	Labels for tweets (positive, negative, neutral).

**Table 3:** Feature description of the Amazon dataset.

Features	Description
ReviewerID	ID of the reviewer
ASIN	ID of the product
Reviewer name	Name of the reviewer
Helpful	Helpfulness rating of the review
Review text	Text of the review
Overall	Rating of the product
Summary	Summary of the review.
UnixReviewTime	Time of the review (unix time).
ReviewTime	Time of the review (raw)

### 3.2 Text preprocessing

The preprocessing step is essential in the sentiment analysis process. It transforms text into a format suitable for machine learning algorithms [28]. The preprocessing of text includes removing retweets because duplicate tweets might skew word frequency and increase the amount of space needed for running the experiment. In the next step, URLs should be removed since they have no meaning and won’t affect sentiment. Removing punctuation, emojis, non-alphanumeric characters, and stop words is critical because they are not helpful for analysis, and in tokenization, the entire text or paragraph is divided into smaller units, known as tokens [29]. Finally, the lemmatization process removes inflectional endings and returns the base or dictionary form of words, and the stemming process reduces words into word stems because some of the words might not be proper in the language. The WordNetLemmatizer lemmatization and PorterStemmer stemming were used for this study. Table 4 shows some examples before and after the preprocessing step from Amazon musical instrument reviews.

**Table 4:** Some examples of Amazon reviews dataset.

Reviews	Reviews after preprocessing
Not much to write about here, but it does exac. . .	much write exactli suppos filter pop sound rec. . .
The product does exactly as it should and is q. . .	product exactli quit affordablei realiz doubl. . .
The primary job of this device is to block the. . .	primari job devic block breath would otherwis. . .
Nice windscreen protects my MXL mic and preven. . .	nice windscreen protect mxl mic prevent pop th. . .

### 3.3 Feature extraction

**Fig 4:**
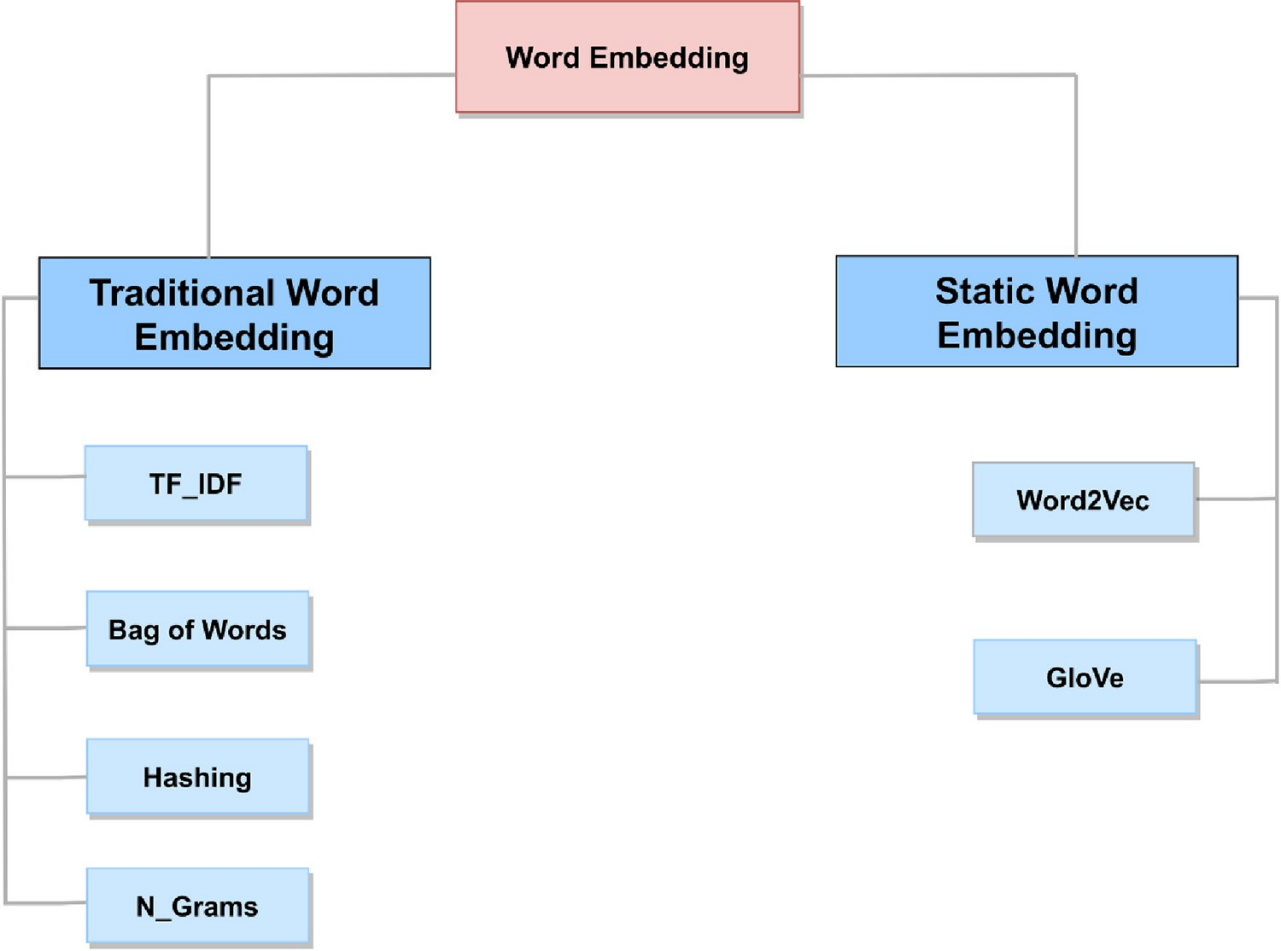
The general structure of the word representation models.

In this study, the main contribution is the extraction of important features from datasets. The process of feature extraction holds significant importance in text processing as it effectively decreases the dimensionality of the feature space by selectively emphasizing the crucial aspects. Hence, in this work, we employed six different feature extraction methods, including Bow, TF-IDF, n-grams with a range of (1,2) which includes both unigrams (individual words) and bigrams (pairs of consecutive words), global vector for word representation (GloVe), hashing vectorizer (HV), and word2vec, to extract features from the datasets, as shown in Fig 4. The selection of these specific feature extraction techniques is based on their established effectiveness in sentiment analysis tasks and their ability to capture different aspects of text data [29]. The chosen feature extraction techniques can improve classification or prediction accuracy and maximize the utility and relevance of the feature extraction process, leading to more meaningful and impactful outcomes.

#### 3.3.1 TF-IDF

This method is derived from language modeling theory. According to the theory, words in a text can be divided into two categories based on their eliteness: those with eliteness and those without. Its calculation is based on a combination of two metrics, one of which measures how many times a word appears in a collection of documents, and the other measures the word’s inverse document frequency. In a document, term frequency (TF) counts the number of times words appear, and inverse document frequency (IDF) is a method that helps distinguish and classify documents easily by giving importance or weightage to words that are unique to a certain set of documents [30]. Words in the document with high or low-frequency terms are given more weight by the IDF. Combining TF and IDF is known as TF-IDF. According to Eq (1), the mathematical representation of the weight of a term in a document by the TF-IDF method.


$$W(d,t)=TF(d,t)*log(\frac{N}{df(t)})$$ (1)

In this equation, N denotes the number of documents, and df (t) indicates how many documents contain the term t in the corpus. The initial term introduces an enhancement to recall, while the subsequent term contributes to precision. Although TF-IDF endeavors to address the issue of frequently occurring terms in a document, it is not without its constraints; for instance, when each word is displayed independently as an index, it is incapable of considering word similarity. However, TF-IDF vectors exhibit superior accuracy compared to alternative methodologies.

#### 3.3.2 BOW

It is one of the simplest feature extraction model categories, and it does not take the order of the words into account. This model is a method of encoding text data. It is simple to use and learn, and it has proven to work effectively for document classification and language modeling. There are some limitations of BOW models like sparsity, and the complete disregard for word order ignores the context, which in turn ignores the semantics of the words used in the document [31]. Three steps describe how this model works: the first step is text tokenization, then tokenizing each sentence into words and counting how many times each word appears in each sentence, and finally, constructing the model by creating a vector to identify If the word is frequently used, it might be set to 1, otherwise 0 and generate the output.

#### 3.3.3 N-Grams

The n-gram technique consists of any sequence of n-words that occur “in that order” in a text set. This technique was the first to attempt to impose a window to capture the ordering among words. The n-gram method ignores individual words and instead focuses on multiword tokens and their ordering within the context window. The N-gram does not necessarily capture contextual information, but it is effective at capturing word ordering among words. When these words appear together, they may have an entirely different meaning than when they appear separately. This model is relatively easy to obtain, and a manageable-sized vector can be used to represent the text [32]. In this study, the n-gram with a range of (1, 2) is used, and this range refers to a combination of unigrams (single words) and bigrams (pairs of consecutive words) in a given text.

#### 3.3.4 Hashing vectorizer

In the hashing vectorization (HV) method, collections of review text are transformed into a matrix of token occurrences. A hashing vectorizer returns the account for every token in the document, so it is no different from a regular BOW model in terms of how text features are turned into a numeric representation. However, hashing vectorizers have the following advantages: they scale better with large document sets and work well with batch processing [33]. The limitation is the potential for hash collisions, and the larger feature space can introduce additional computational overhead, leading to longer training times.

#### 3.3.5 Static word embedding

Word embeddings are numerical representations of words or phrases that depict the relationships between them in a multidimensional space as well as their semantic meaning. These representations are typically learned from large amounts of text data using neural networks. There are some key characteristics of word embedding such as similar words having the same embedding, values and each word having a distinct word embedding or vector, which is only a list of numbers for each word. This study uses the word2vec model and the glove model, two of the most popular algorithms for word embeddings. The first model is the Word2Vec Model was first introduced by [34], is popular and widely used in learning word embeddings from raw text. Based on the idea of distributed representation of words, word2vec (word embeddings) uses a shallow neural network to learn word embeddings and predict the relation between every word and its context words. With this method, relevant information from the texts is captured, resulting in good results.

In word2vec, SG (skip-gram) and CBOW (Continuous Bag-of-Words) algorithms are used to produce word vectors [34]. The SG model is used to store semantic and syntactic information about sentences. In this study, the SG model is implemented with a vector size of 100, which means that each word will be represented by a vector of length 100, and a window size of 5, which shows the maximum distance between the current and predicted word within a sentence. The choice of the value of the window parameter balances between capturing local context and capturing broader semantic relationships, and the vector provides a good balance between capturing semantic information and computational efficiency. The aim of this model is to maximize the classification of words based on other words in the same sentence.

The GLOVE (Global Vectors for Word Representation) method has been developed by [35]. This method is used for producing word embeddings and is an unsupervised procedure. A meaningful space is constructed for the words, in which the distance between words correlates with semantic similarity. The global word cooccurrence matrix is aggregated from a corpus for training purposes. As a result, the resulting representations of the word exhibit interesting linear substructures in vector space. In this model, a large corpus of data has been used to train it [36]. The model is not able to capture out-of-vocabulary words from the corpus and consumes a great deal of memory during storage. It is effective and scalable for huge corpora because it combines latent semantic analysis and CBOW. We perform experiments using a vector embedding dimension of 300.

### 3.4 Synthetic minority over-sampling technique

The distribution of positive, neutral, and negative polarities in the datasets in this study is unbalanced. This imbalanced data may have a significant negative impact on the machine learning models’ performance because it may tilt the decision surface in favor of the majority class. The oversampling approach is used to solve the issue of class imbalance. This approach works by increasing the size of the data, which creates more features for model training and could be helpful to enhance the model’s accuracy. In this study, we use the synthetic minority oversampling (SMOTE) method for oversampling. The SMOTE is a state-of-the-art method proposed by [37, 38]. This method was selected because it avoids information loss, is simple to interpret and implement, and helps to solve the overfitting issue for unbalanced datasets. Randomly, SMOTE selects the smaller classes and finds their K-nearest neighbors. Based on the K-nearest neighbor for each selected sample, a new minority class is constructed [39]. With 70:30 ratios, the data is divided into training and testing sets after the oversampling process.

### 3.5 Machine learning model

The proposed ensemble classifier was trained on the training set for classifying the sentiments in the datasets and evaluated on the test data. The ML algorithm used in this work is a random forest classifier. This model was chosen for this study because it helps to avoid overfitting, provides a measure of feature importance, and produces a reasonable prediction without adjusting hyperparameters. This is a supervised ML algorithm that is used for regression and classification purposes and belongs to the ensemble learning family [40]. In the random forest model, decision trees are constructed from datasets and create a forest made of trees. A random forest classifier consists of the following steps: The first step is to select random data samples from the available dataset. For each selected data sample, a decision tree is constructed, and a prediction value is extracted from each decision tree. For node splitting, the Gini coefficient method is applied as follows:

$$Gini(D)=1-\sum_{i=1}^{n}p_{i}^{2}$$ (2)

Where D represents the dataset and Pi represents the probability of decision classes appearing in D. After obtaining prediction values from each decision tree, a voting method is applied. The final prediction result is selected based on the prediction value with the most votes [41]. In order to increase accuracy, RF was implemented with n-estimators equal to 100, which indicates how many trees contributed to the prediction. To decrease the probability of the decision tree overfitting, the `max_depth’ setting is set to 5, which shows that every decision tree can go to a maximum of five levels.

### 3.6 Performance measures

To examine the performance of the suggested model using different feature extraction techniques, we used several standard performance measures. Specifically, we used recall, accuracy, precision, and F1-measure. To calculate all four metrics, machine learning models can be visualized by using a confusion matrix [42, 43]. The elements of this matrix are False Negative (FN), True Positive (TP), False Positive (FP), and True Negative (TN). The performance evaluation of classifiers is made according to the following formulas:

$$Accuracy:\;\frac{TP+TN}{TP+TN+FP+FN}$$ (3)

$$Precision:\;\frac{TP}{TP+FP}$$ (4)

$$Recall:\;\frac{TP}{TP+FN}$$ (5)

$$F1‐Measure:\;2*\frac{Precision*Recall}{Precision+Recall}$$ (6)

## 4. Experimental results

We conducted the experiments on Google Colab, a cloud-based graphical processing unit (GPU)-based platform offered by Google Inc. The classification algorithm was implemented using the Scikit-learn library. Due to the moderate size of the dataset, ML algorithms are used rather than deep learning algorithms for classification. We conducted experiments using a set of datasets that are commonly used in sentiment analysis by applying a random forest classifier using different word representation models and based on the parameters shown in Table 5.

**Table 5:** The parameters tuned with respect to the random forest model.

Parameters	Values
n_estimators	100
Criterion	Gini
max_depth	5
max_features	sqrt
random_state	42

It is observed that the sentiment classes in the datasets are imbalanced, so the SMOTE technique is applied. For the two datasets, a total of 70% is used for the training process, and the other 30% is used for testing using the random forest model as shown in Table 6. The performance of the random forest algorithm is evaluated on different metrics such as recall, precision, accuracy, and F1-measure.

**Table 6:** The total size and size of (train/test) of the datasets.

Dataset	Total size	Training Set size	Testing Set size
Twitter US airlines	14640	10248	4392
Amazon musical instrument reviews	10261	7183	3078

Computational efficiency is calculated by using the training time which is the time it takes to train the model, and the prediction time which is the time it takes to predict the labels for a new set of instances after each feature extraction. A comparison of all the feature extraction methods on the Twitter dataset is shown in Table 7, where the TF-IDF and HV methods achieve the highest accuracy, but the TF-IDF is much faster than others. The n-gram has the lowest accuracy, but it also has a low training time.

**Table 7:** Performance and time of the random forest classifier on a Twitter dataset.

Feature extraction	accuracy	Precision	Recall	F1-measure	Training time	Prediction time
TF_IDF	96	95	96	95	11.285836	0.497233
N_Gram	86	87	86	86	13.926802	0.541020
BOW	87	87	87	87	16.031671	0.535141
Hashing Vectorizer	96	96	96	96	79.441338	0.809710
Word2Vec	93	93	93	93	19.753669	0.214723
Glove	92	92	92	92	35.825151	0.180461

A comparison of all the feature extractions on the Amazon reviews dataset is shown in Table 8. The TF-IDF achieves the highest precision, accuracy, recall, and F1-measure. It also has the lowest training time.

**Table 8:** Performance and time of the random forest classifier on the Amazon dataset.

Feature extraction	Accuracy	Precision	Recall	F1-measure	Training time	Prediction time
TF_IDF	99	99	99	99	8.738268	0.199666
N-gram	89	93	92	92	10.649860	0.217366
BOW	90	91	90	90	10.967826	0.209918
Hashing Vectorizer	98	98	99	98	62.155688	1.326388
Word2Vec	96	96	96	96	8.990855	0.101492
Glove	97	97	97	97	48.304039	0.231222

**Fig 5:**
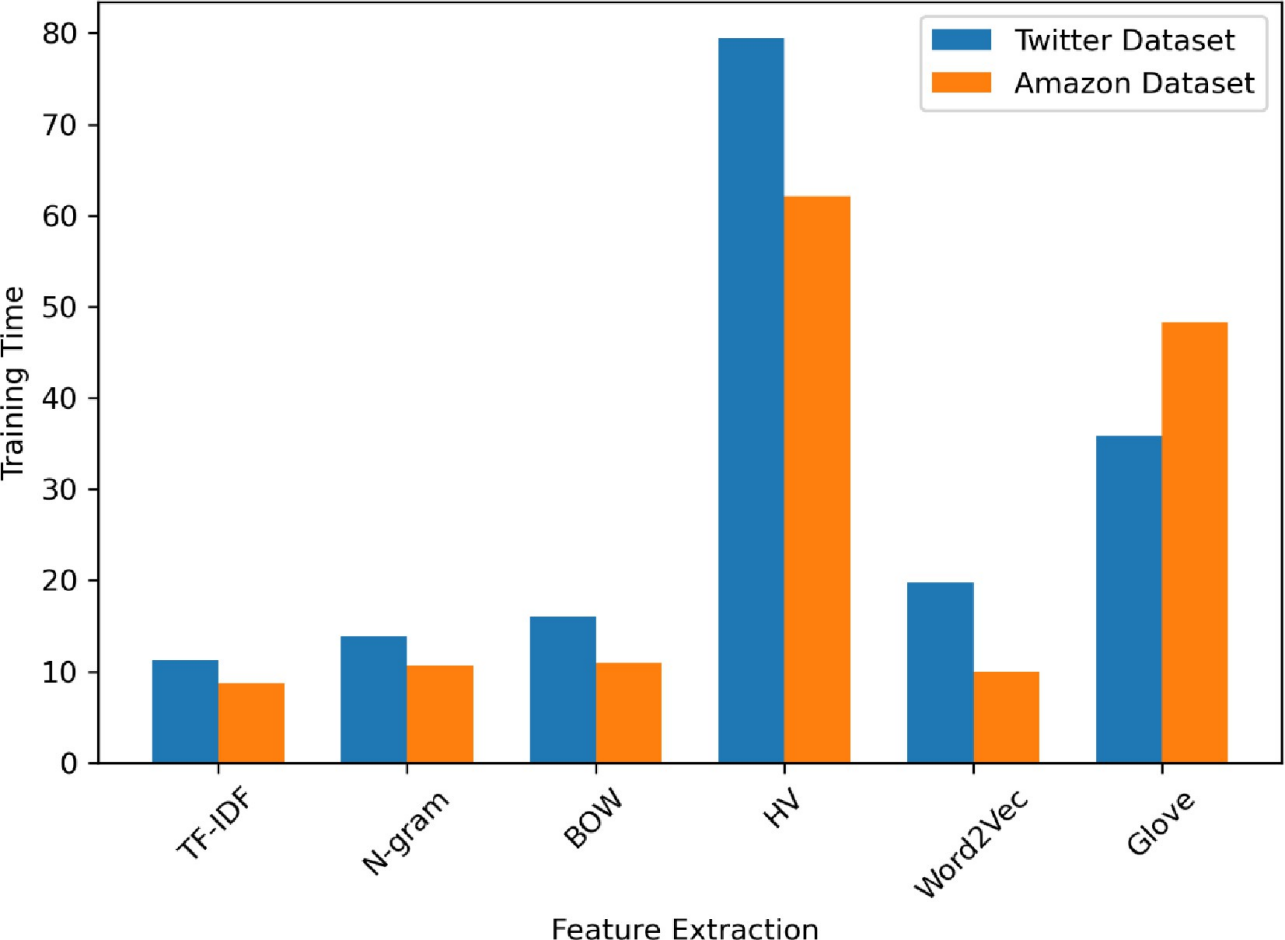
The training time of the datasets.

Fig 5 displays a comparison of the training time for the proposed model following the dataset’s feature extraction. The HV method requires significantly more training time compared to other methods.

**Fig 6:**
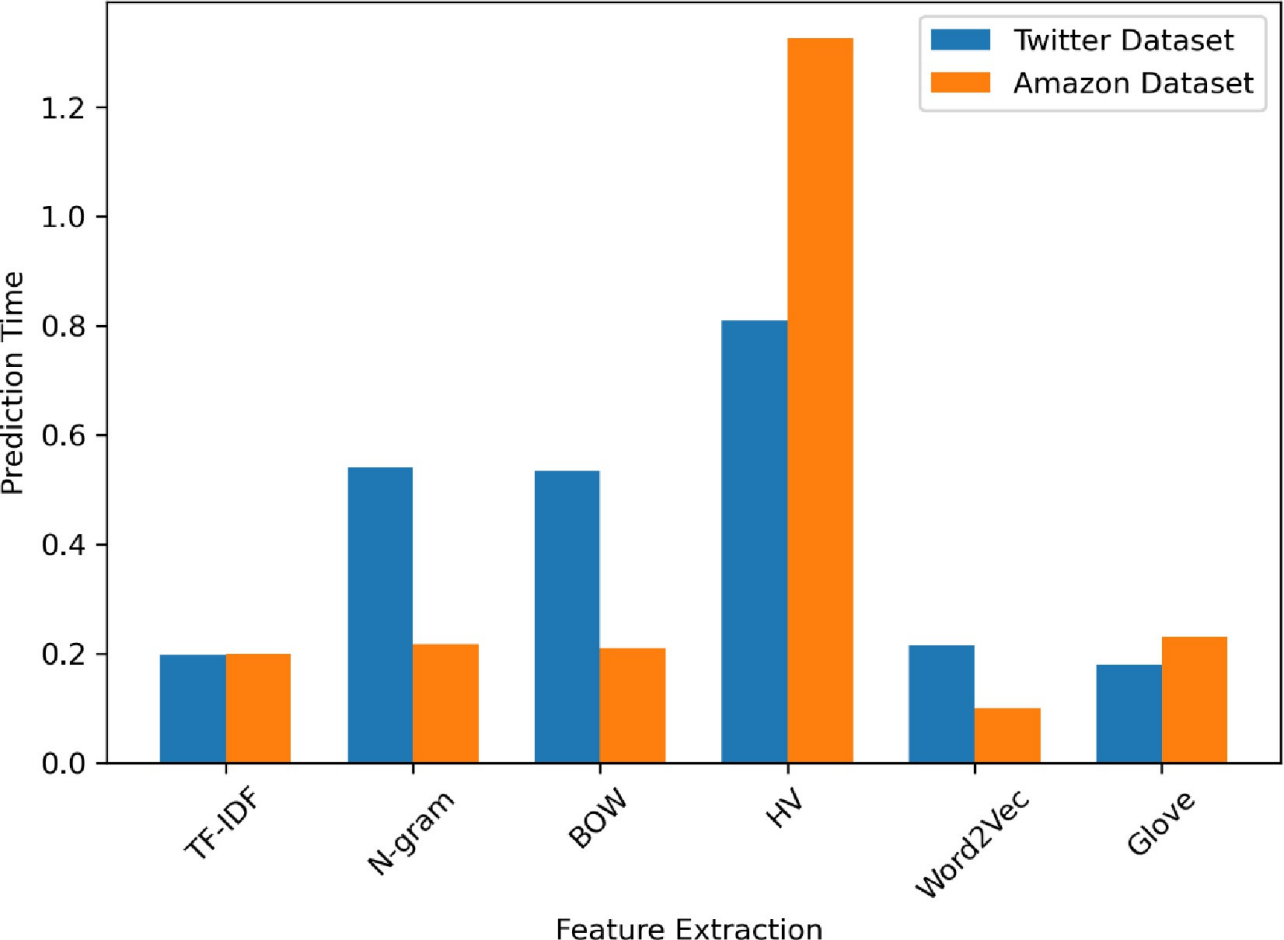
The prediction time of the datasets.

**Fig 7:**
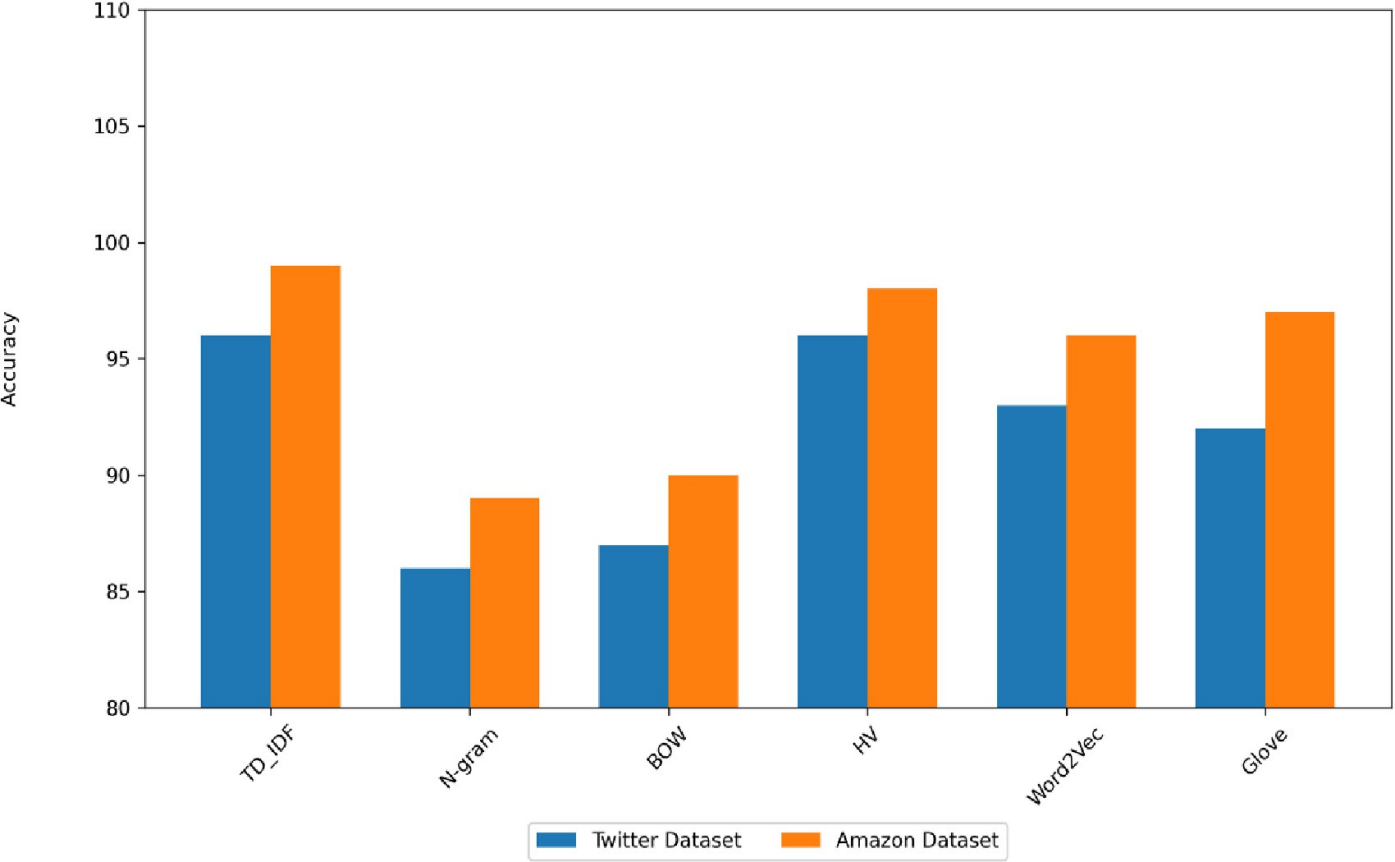
The accuracy of the datasets.

Fig 6 displays the testing time for the proposed model following the dataset’s feature extraction. The HV method takes a longer prediction time, followed by the n-gram and Bow methods. Fig 7 displays the proposed model’s accuracy. The highest accuracy values of the proposed model on the datasets for TF-IDF and HV methods.

## 5. Discussion

In this section, we will have an overall discussion of the experimental results from the previous section. It has been noted that all the feature extraction methods performed well, with high accuracy and balanced precision, recall, and F1-measure, so the model’s performance is not skewed by the majority class and the model can generalize well to all classes. This suggests that the other methods are also capable of extracting important features from the text data.

From Fig 6, the comparison between the outcomes proves that the performance of the model is the highest after the TF_IDF and HV methods for both datasets. The TF-IDF achieves an accuracy of 99% with the Amazon dataset and 96% with the Twitter dataset. The performance of the model is improved, especially on TF-IDF vectorization because the model can benefit from the ability of this extractor to focus on important and discriminative terms while down-weighting common and less informative terms.

BOW performs similarly to the n-gram method, with slightly lower accuracy and F1-measure in which the accuracy of BOW is 90% on the Amazon dataset and 87% on another dataset. The BOW method shows consistent precision and recall across both datasets, indicating that it maintains a good balance between correctly identifying sentiments. From Fig 4 the training time was relatively fast in the TF-IDF but the training time of HV is the longest in both datasets due to the hashing process and the potential for hash collisions. The Word2Vec and GloVe models have slightly lower accuracy than TF-IDF, but Word2Vec is much faster to train than the GloVe model, especially for the Amazon reviews dataset. From Fig 5 the prediction time of the HV method is the longest on both datasets but the prediction time of TF-IDF is relatively the same on both datasets.

Overall, the TF-IDF extractor provides a good balance between performance across all evaluation metrics and computational efficiency. Thus, it is important to consider the trade-off between training time and the specific requirements of the sentiment analysis task when choosing a feature extraction method because some techniques may yield higher accuracy, but the training time becomes too long, and this may not be practical for real-time sentiment analysis applications.

Our experiment has shown that selecting the right feature extraction method has a significant impact on the performance of an ML algorithm, which means that rather than spending a lot of time optimizing a specific classifier, it might be worthwhile to spend more time choosing the right feature extraction method. Also, the impact is on business organizations that may be able to detect negative reviews more efficiently. In a short period of time, business organizations can learn about customer demand after inspecting negative reviews, and they can reshape their products and policies accordingly.

Although we have shown successful feature extraction-based sentiment analysis and ML, there are several limitations to this work that could be explored in the future: this study is based on only English-language reviews that were analyzed and another limitation is that we have only tested the random forest model in our experiments.

## 6. Conclusion

In the last few years, feature extraction and machine learning have become more popular for analysis and prediction. The effectiveness of sentiment analysis on social media is studied in this research using six distinct feature extraction techniques, and the key findings are discussed. So, in this work, there are two different datasets from diversified social media platforms to evaluate the performance of the suggested model. A data preprocessing stage is executed on the dataset to remove several superfluous symbols and then employ feature extraction with the SMOTE technique. A state-of-the-art ML algorithm is used to train the extracted features, namely the random forest algorithm. After each feature extraction, the ML algorithm’s performance is evaluated.

On both datasets, the random forest offers the highest accuracy with TF-IDF and fewer training and prediction times than others. The results indicate that the choice of suitable methods for feature extraction plays a crucial role in determining the effectiveness of sentiment analysis tasks, with some techniques performing better than others. These findings have important implications for practitioners and researchers working in the field of sentiment analysis. They suggest that careful consideration should be given to the choice of feature extraction techniques when developing sentiment analysis models for social media.

In future studies, the analysis can be expanded to include other languages, such as Arabic, and can explore other machine learning models, such as deep learning models or transformers, to see if they can improve the accuracy of sentiment analysis on imbalanced datasets with different feature extraction techniques. Additionally, we can use hybrid feature extraction techniques to explore the impact of this improvement on the performance of the sentiment analysis classification. Finally, we intend to apply our method to more recent datasets.

## Supporting information

S1 File The file contains the data and supporting tables. (DOCX)
