# Supplementary material for: Enhancing machine learning-based sentiment analysis through feature extraction techniques
Source: PLoS One. 2024 Feb 14;19(2):e0294968. doi: 10.1371/journal.pone.0294968 (PMC10866497; doi:10.1371/journal.pone.0294968)
Supplement: S1 File — (DOCX) [file pone.0294968.s001.docx]

Supporting Information for

“Enhancing Machine Learning-Based Sentiment Analysis through Feature Extraction Techniques”

Noura A. Semary^1^, Wesam Ahmed^1,2^, Khalid Amin^1^, Paweł Pławiak^3,4*^, Mohamed Hammad^1,5*^

*** Correspondence @**

**S1 File. The file contains the data and supporting Tables.**

1. **Data:**

| **Database Name** | **Link** |
| --- | --- |
| Twitter US Airline | https://www.kaggle.com/datasets/crowdflower/twitter-airline-sentiment |
| Amazon Musical Instruments Reviews | https://www.kaggle.com/datasets/eswarchandt/amazon-music-reviews |

## 2. Supplementary Tables

**Table 1. Literature survey of sentiment analysis.**

| Ref. | Dataset | Feature Extraction | Model | Results |
| --- | --- | --- | --- | --- |
| Ahmed et al.  (2023) | Collected news articles | TF-IDF | NB | Accuracy = 89.30% |
| Gaur et al. (2023) | Twitter sentiment 140 | TF-IDF | NB | Accuracy = 84.44% |
| Qi and Shabrina.  (2023) | Collected tweets about COVID-19 | TF-IDF, and Word2Vec | MNB, SVC, RF, Vader, and Textblob | SVC with TF–IDF outperforms others |
| Al sari et al.  (2022) | Instagram, Snapchat, and Twitter datasets | Unigrams | MLP, NB, RF, SVM, and voting | RF algorithm achieved a high level of accuracy |
| Mukherjee et al.  (2021) | Amazon reviews | TF-IDF | MNB, SVM,and ANN | ANN + Negation classifier performs the best |
| Noori.(2021) | Customer reviews | TF-IDF | NB, SVM, DT, and KNN | Best accuracy reported for DT |
| Zahoor and Rohilla .  (2020) | Collected tweets about different events | N-gram | NB, SVM, RF, and LSTM | NB outperforms others on most datasets |
| Ref. | Dataset | Feature Extraction | Model | Results |
| Samuel et al.  (2020) | COVID-19 tweets | N-gram | NB and LR | NB outperforms LR |
| Kumar et al.  (2020) | Book reviews | BOW, and Word2Vec | NB, ME, and SVM | SVM has the highest accuracy = 78% |
| Kermani et al.(2019) | Twitter  datasets | TF-IDF | SVM, MNB and hybrid algorithm | The hybrid method yields a better classification |

**Table 2. Some examples of Amazon reviews dataset.**

| Reviews | Reviews after preprocessing |
| --- | --- |
| Not much to write about here, but it does exac... | much write exactli suppos filter pop sound rec... |
| The product does exactly as it should and is q... | product exactli quit affordablei realiz doubl ... |
| The primary job of this device is to block the... | primari job devic block breath would otherwis ... |
| Nice windscreen protects my MXL mic and preven... | nice windscreen protect mxl mic prevent pop th... |

**Table 3. Performance and time of the random forest classifier on a Twitter dataset.**

| **Feature extraction** | **accuracy** | **Precision** | **Recall** | **F1-measure** | **Training time** | **Prediction time** |
| --- | --- | --- | --- | --- | --- | --- |
| TF_IDF | 96 | 95 | 96 | 95 | 11.285836 | 0.497233 |
| N_Gram | 86 | 87 | 86 | 86 | 13.926802 | 0.541020 |
| BOW | 87 | 87 | 87 | 87 | 16.031671 | 0.535141 |
| Hashing Vectorizer | 96 | 96 | 96 | 96 | 79.441338 | 0.809710 |
| Word2Vec | 93 | 93 | 93 | 93 | 19.753669 | 0.214723 |
| Glove | 92 | 92 | 92 | 92 | 35.825151 | 0.180461 |

**Table 4.** **Performance and time of the random forest classifier on the Amazon dataset.**

| **Feature extraction** | **Accuracy** | **Precision** | **Recall** | **F1-measure** | **Training time** | **Prediction time** |
| --- | --- | --- | --- | --- | --- | --- |
| TF_IDF | 99 | 99 | 99 | 99 | 8.738268 | 0.199666 |
| N-gram | 89 | 93 | 92 | 92 | 10.649860 | 0.217366 |
| BOW | 90 | 91 | 90 | 90 | 10.967826 | 0.209918 |
| Hashing Vectorizer | 98 | 98 | 99 | 98 | 62.155688 | 1.326388 |
| Word2Vec | 96 | 96 | 96 | 96 | 8.990855 | 0.101492 |
| Glove | 97 | 97 | 97 | 97 | 48.304039 | 0.231222 |
